# Exploiting azide–alkyne click chemistry in the synthesis, tracking and targeting of platinum anticancer complexes

**DOI:** 10.1016/j.cbpa.2019.12.001

**Published:** 2020-04

**Authors:** Nicola J. Farrer, Darren M. Griffith

**Affiliations:** 1 Chemistry Research Laboratory, University of Oxford, 12 Mansfield Road, Oxford, OX1 3TA, UK; 2 Department of Chemistry, RCSI, 123 St. Stephens Green, Dublin 2, Ireland; 3 SSPC, Synthesis and Solid State Pharmaceutical Centre, Ireland

**Keywords:** Platinum, Anticancer, Azide, Alkyne, Triazole, CuAAC, SPAAC, Cycloaddition, iClick, Synthesis, Target, Track, Functionalise, Analyse

## Abstract

Click chemistry is fundamentally important to medicinal chemistry and chemical biology. It represents a powerful and versatile tool, which can be exploited to develop novel Pt-based anticancer drugs and to better understand the biological effects of Pt-based anticancer drugs at a cellular level. Innovative azide–alkyne cycloaddition–based approaches are being used to functionalise Pt-based complexes with biomolecules to enhance tumour targeting. Valuable information in relation to the mechanisms of action and resistance of Pt-based drugs is also being revealed through click-based detection, isolation and tracking of Pt drug surrogates in biological and cellular environments. Although less well-explored, inorganic Pt-click reactions enable synthesis of novel (potentially multimetallic) Pt complexes and provide plausible routes to introduce functional groups and monitoring Pt-azido drug localisation.

## Highlights

• Click chemistry is integral to the chemical biology of Pt anticancer complexes.
• The Cu(I)-catalysed [3 + 2] azide–alkyne cycloaddition (CuAAC)/strain-promoted [3 + 2] azide–alkyne cycloaddition (SPAAC) enable the functionalisation of Pt drug candidates.
• CuAAC/SPAAC aid the detection, isolation and tracking of Pt drugs.
• CuAAC/SPAAC facilitate the development of novel Pt triazole–based complexes.

## Graphical abstract

**Figure:**
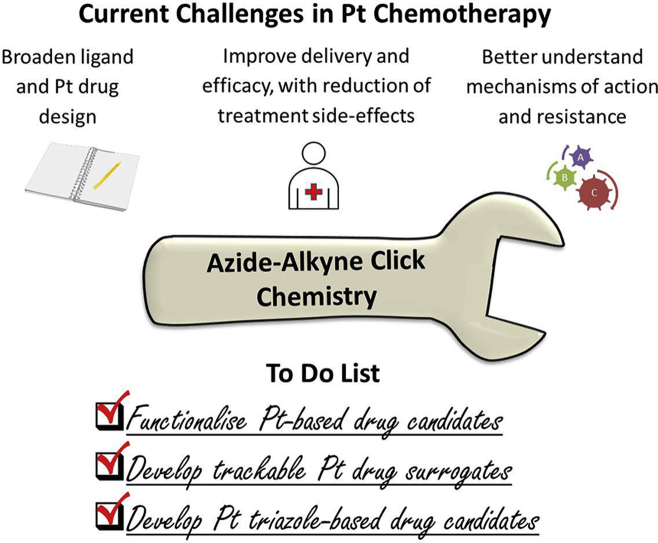

## Introduction

**Figure 1:**
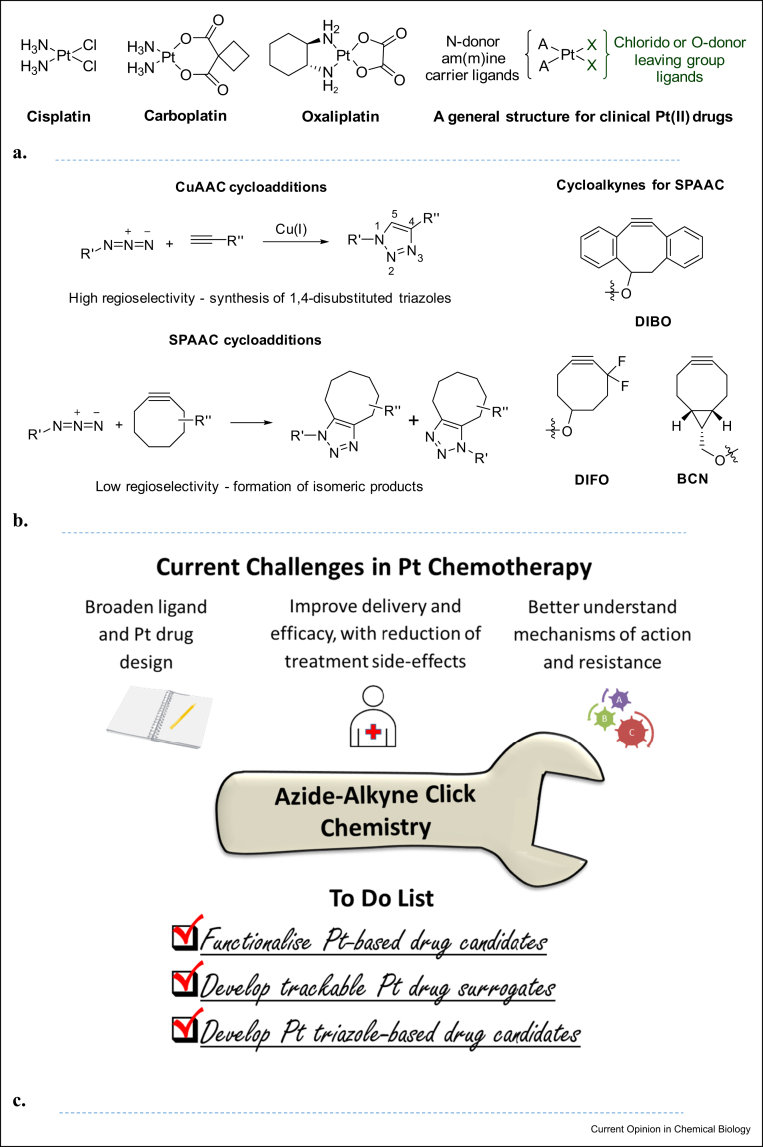
**Pt drugs, azide-alkyne cycloadditions and article focus.(a)** Structures of three clinically approved Pt(II)-based anticancer drugs. **(b)** General schemes for azide–alkyne click reactions and representative cycloalkynes used for SPAAC, where DIBO is dibenzocyclooctyne, DIFO is difluorocyclooctyne and BCN is bicyclononyne, N.B. SPAAC with fuctionalised cycloalkynes can lead to the formation of regioisomers (e.g. DIBO derivatives and nonchiral azide), enantiomers (e.g. BCN and nonchiral azide) or diastereoisomers (e.g. BCN and chiral azide) **(c)** Outline of article focus. SPAAC, strain-promoted [3 + 2] azide–alkyne cycloaddition.

Of the cancer patients who are treated with chemotherapy, around 50% receive a Pt(II)-based medicine such as cisplatin, carboplatin or oxaliplatin (Figure 1a) [1]. The primary mechanism of action of cisplatin and carboplatin results from their ability to cross-link nuclear DNA; the Pt-DNA adducts interrupt transcription, generate DNA damage responses and ultimately induce apoptosis. Pt(II) anticancer drugs also react with a range of other nucleophiles, including RNA, mitochondrial DNA and proteins [2,3]. Oxaliplatin is used clinically to treat stage III colorectal cancer and exhibits a fundamentally different cytotoxic profile to cisplatin and carboplatin. Although DNA platination does occur, other effects including induction of immunogenic cell death [1,4] and ribosome biogenesis stress are thought to dominate the anticancer mechanism of action of oxaliplatin [5].

The clinical effectiveness of Pt anticancer agents is hampered by toxic side effects and both intrinsic and acquired resistance [6]. Therefore, there has been a continued drive to develop novel classes of more effective and better-tolerated Pt(II) and Pt(IV) drug candidates, as well as to better understand the precise cellular and immunological effects of Pt complexes [7]. The development of innovative techniques to synthesise, label and track Pt(II)- and Pt(IV)-based complexes is anticipated to greatly aid this enterprise. Click chemistry is widely used throughout synthetic chemistry and biology, showing tremendous versatility, whilst being atom efficient and in some cases, bio-orthogonal.

The Cu(I)-catalysed [3+2] azide–alkyne cycloaddition (CuAAC), Figure 1b, is synonymous with click chemistry [8,9]. Reaction of an azide with a terminal alkyne generates the corresponding 1,4-disubstituted 1,2,3-triazole with excellent selectivity and in high yield. CuAAC has been successfully and routinely used in the syntheses of 1,2,3, triazoles,[10∗] many of which have interesting biomedical applications [11, 12, 13]. Triazoles are attractive pharmacophores which can potentially intercalate, participate in hydrogen bonding and can act in some respects as a substitute for amides [14]. CuAAC has also been used to develop triazole-based ligands[10∗,^15^], and for chemical conjugations including for labelling in biological systems, though reactions in living systems are limited by Cu(I)-associated toxicity [16]. Strain-promoted [3+2] azide–alkyne cycloadditions (SPAACs), Figure 1b, better fulfil the criteria required for bio-orthogonal chemistry [16], which is integral to chemical biology [17∗].

The widespread popularity of click chemistry is certain. This article will outline, Figure 1c, how click chemistry, specifically azide–alkyne cycloaddition, is an important chemical tool for:

• the functionalisation of Pt-based drug candidates
• the development of trackable Pt drugs
• the development of Pt triazole drug candidates.

## Functionalisation

**Figure 2:**
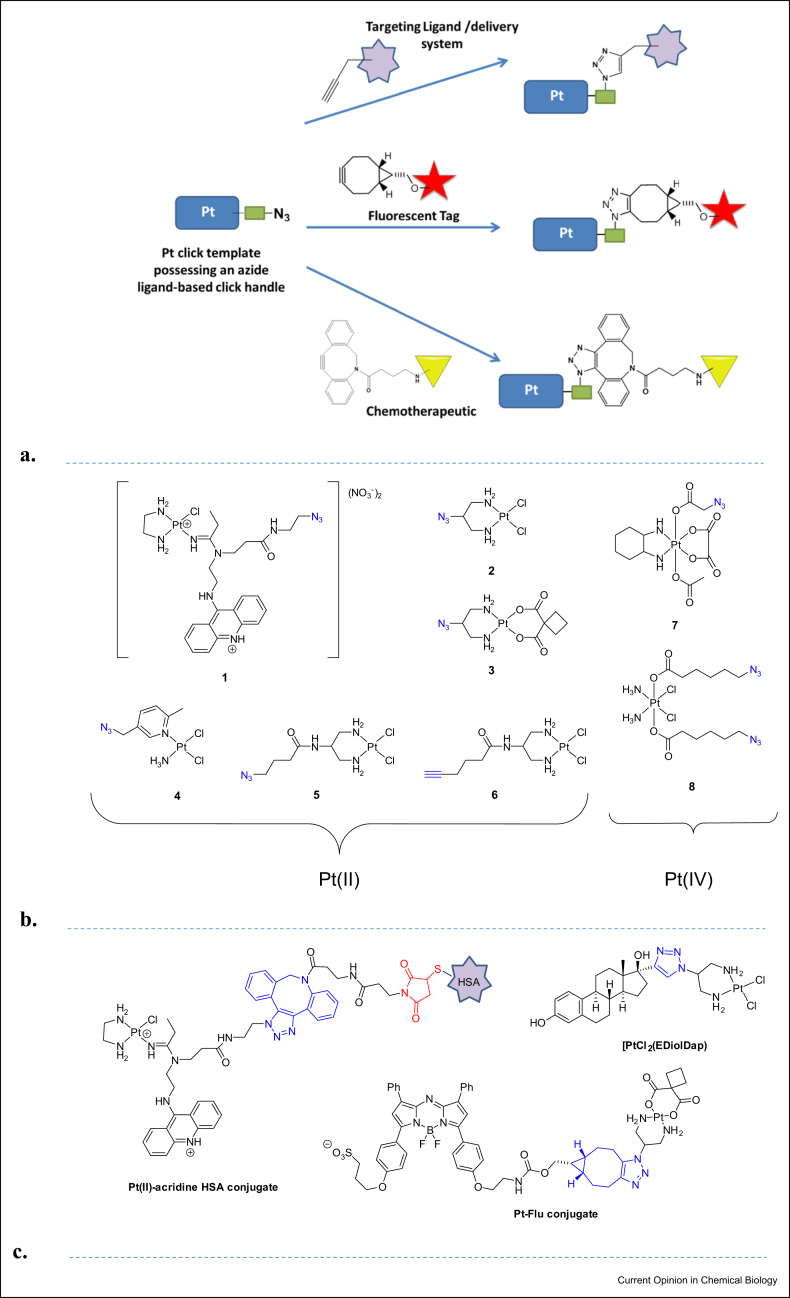
**Pt ligand-based click platforms and conjugates. (a)** Representative Pt ligand-based click platform for the functionalisation of Pt complexes. **(b)** Structures of Pt ligand-based click templates; azide modified Pt(II) acridine complex **1** [21,22], *cis*-[Pt(2-azidopropane-1,3-diamine)Cl_2_] **2** [23,24], picazoplatin **3** [25], [Pt(2-azidopropane-1,3-diamine)(CBDCA_-2H_)] **4** [20∗,^23^] azidoplatin **5** and (f) *cis*-[Pt(2-(5-hexynyl)amido-1,3-propanediamine)Cl_2_] **6** [26] *cis,cis,trans*-[Pt(DACH)(Ox)(OAc)(OAc-N_3_)] **7** [27] and Platin-Az **8** [28]. Figure 2 (c) Structures of Pt(II) human serum albumin (HSA) conjugate,[18∗] Pt(II) estrogendiol (EDiol) conjugate [19] and Pt(II) fluorophore (Flu, NIR-AZA) conjugate [20∗].

Click chemistry represents an excellent conjugation strategy for the functionalisation of Pt complexes with, for example, targeting moieties/delivery systems, fluorescent reporters and secondary chemotherapeutics, Figure 2a. A variety of Pt(II) and Pt(IV) click templates have been developed to date, which possess alkyne or azide ligand-based click handles, Figure 2b.

### Targeting agents

Selective coupling of chemotherapeutics to human serum albumin (HSA), the most abundant blood serum protein, has proved to be a successful strategy for enhanced tumour targeting because HSA can act as a long-circulating delivery vehicle which accumulates passively in the tumour tissue. Yao et al [18∗] successfully used a DIBO-maleimide linker for cysteine-specific maleimide Michael addition chemistry and strain-promoted [3 + 2] azide–alkyne cycloaddition (SPAAC) chemistry to conjugate a cytotoxic electrophilic Pt-acridine anticancer agent to HSA, Figure 2c.

Steroid hormone receptors are well-known targets for cancer chemotherapeutics as many are overexpressed in cancer cells. Kitteringham et al. [19] used CuAAC to synthesise a 1,4 disubstituted 1,2,3 triazole and oestrogen-based ligand, EDiolDap and the corresponding Pt(II) triazole-linked oestrogen complexes [PtCl_2_(EDiolDap)] and [Pt(CBDCA)(EDiolDap)], respectively, Figure 2c. Both complexes exhibited superior activity as compared with cisplatin in 2D cell culture and showed *ca.* 30-fold higher activity, in terms of IC_50_ values, against ER+ cancer cells (cervical, breast and ovarian) in comparison with the reference ER-colon cancer line. [PtCl_2_(EDiolDap)] and [Pt(CBDCA)(EDiolDap)] retained their activity in an ovarian 3D spheroid model, reducing the viability of ovarian cancer cell spheroids *ca.* ninefold and fivefold, respectively, in comparison with cisplatin.

### Fluorescent agents

Kitteringham et al.[20∗] also developed a novel carboplatin-like cytoplasmic-trackable near-infrared emitting fluorophore conjugate via SPAAC. Reaction of *cis*-[Pt(2-azidopropane-1,3-diamine) (CBDCA)] and a bicyclo[6.1.0]non-4-yne near-infrared BF_2_ azadipyrromethene (NIR-AZA) fluorophore gave the corresponding clicked Pt–fluorophore conjugate, Figure 2c. The Pt NIR-AZA conjugate was nontoxic and dispersed relatively uniformly throughout the cytoplasm. Inevitably, as for any derivative the properties associated with the preclicked Pt–fluorophore conjugate were strongly influenced by the physical and chemical properties of the organic fluorophore that impact uptake and guide localisation of the complex. It is however still valuable to be able to compare and contrast the proprties of the trackable derivatives with the original drugs in terms of global properties (uptake, efflux *etc*.), and DNA adduct formation and metabolic processing. There is also the potential for the trackable derivative to be developed as a novel drug molecule in its own right, if the biological properties are sufficiently promising.

### Secondary chemotherapeutics

There is a dearth of literature examples where click-based conjugation strategies have been used to link Pt centres to labile secondary chemotherapeutics, likely because of the relative ease of functionalising axial ligands of Pt(IV) hydroxo complexes with carboxylic acid–containing or carboxylic acid–derivatised bioactive molecules. Nonetheless, examples such as *cis,cis,trans*-[Pt(DACH)(Ox)(OAc)(OAc-N_3_)] **7** and Platin-Az **8**, Figure 2b, which contain an axial azide pendant group are anticipated to facilitate Pt(IV) prodrug development via CuAAC and SPAAC functionalisation with additional bioactive ligands including histone deacetylase inhibitors, p53 agonists, alkylating agents and nonsteroidal antiinflammatory agents and so on.

## Trackable Pt-based drugs

Click chemistry has a very important role to play in the isolation of Pt-bound biomolecules and detection of Pt-based drugs in a biological setting. In particular, DeRose and Bierbach have pioneered robust and efficient CuAAC- and SPAAC-based methods (i) for post-treatment analysis of Pt biomolecular interactions,[25,26,29,30∗∗] and (ii) to track Pt-based drugs in cells [21,22]. Numerous clickable fluorescent dyes featuring a variety of click partners including azide, alkyne, dibenzocyclooctyne and bicyclononyne are commercially available. A thorough understanding of molecular properties, the uptake, cellular localisation preferences and efflux of the selected complementary fluorophore click derivative are prerequisites for any study.

### Post-treatment fluorescent analysis

**Figure 3:**
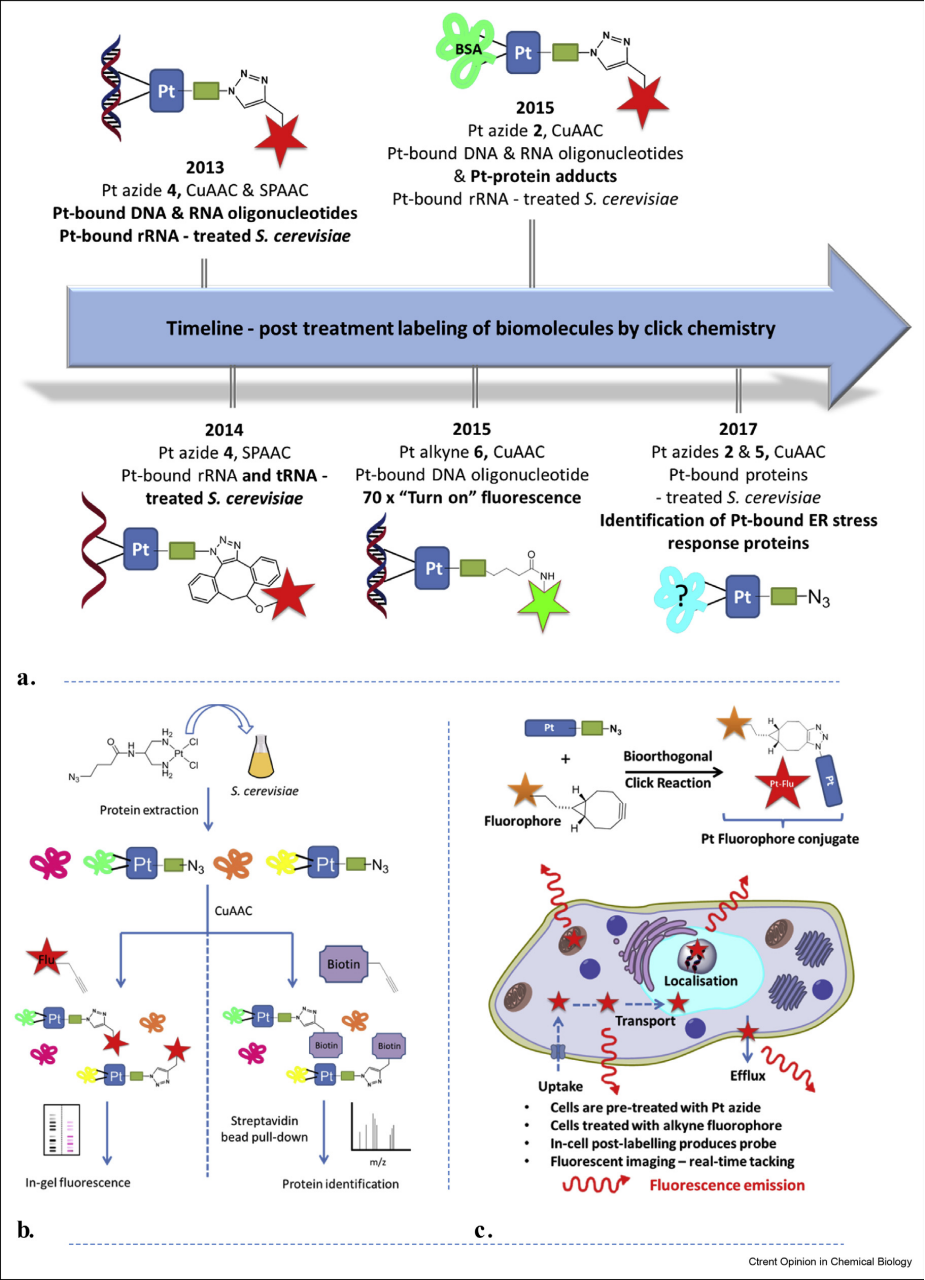
**Post-treatment labelling of Pt-bound biomolecules and real-time tracking of Pt drug surrogates. (a)** Reports to date concerning click chemistry enabled post-treatment labelling of Pt-bound biomolecules. **(b)** Workflow for labelling of cellular Pt-bound proteins; treatment of *S. cerevisiae* with Pt azide complex 5 and protein extraction, followed by CuAAC-enabled labeling of Pt-bound proteins with a fluorescent tag or biotin. Diagram adapted from the previously reported Figure 1 (b) ACS Chem. Biol. 2017, 12 (11), 2737–2745. **(c)** Strategy for click-enabled real-time tracking of Pt drug surrogates. CuAAC, Cu(I)-catalysed [3 + 2] azide–alkyne cycloaddition.

CuAAC and SPAAC click–derivatised Pt(II) complexes have been successfully used for the convenient detection of Pt(II)–DNA, Pt(II)–RNA and Pt(II)–protein adducts in biological media, cell extracts and in yeast cells [24, 25, 26,29, ∗∗30, 31, 32]. A complementary click-functionalized fluorophore is introduced after the Pt click–functionalised complex has bound to its biomolecular targets, Figure 3a.

Significantly recently azidoplatin, Figure 2b (**5**), was used in a novel chemical proteomic method to label and isolate platinated proteins in *S. cerevisiae*.[30∗∗] One hundred and fifty two Pt(II)-bound proteins were identified including multiple proteins implicated in the ER stress response,[30∗∗] highlighting the importance of postlabelling/chemical proteomic strategies in identifying biomolecules implicated in the mechanisms of on- or off-target activity of Pt(II)-based drugs.

CuAAC and SPAAC have been validated as important tools for post-treatment labelling of Pt(II) biomolecular adducts. Future work is likely to focus on labelling Pt adducts extracted from cancer cells, particularly involving high-throughput studies to isolate and identify important Pt(II) protein adducts and related cell–death pathways.

### Track and analyse

In cellulo fluorescent detection of a Pt drug, using confocal microscopy, facilitates tracking of the drug, providing vital information concerning uptake, cellular transport, subcellular/organellular localisation and efflux. Fluorescent detection can be undertaken through in cellulo postbinding bio-orthogonal ligation strategies, Figure 3c.

Use of Pt-based drug derivatives which possess relatively innocent bio-orthogonal click handles tethered to the stable amine carrier ligands enables retention of the essential chemical and biological properties of the parent drug.

The use of click chemistry (CuAAC and SPAAC) has been shown to be a powerful method for mapping the subcellular localisation of post labelled Pt drug surrogates in fixed cancer cells [32,33], including the cell cycle specific localisation [22]. Nonetheless, future developments in the tracking of Pt-based drugs are likely to focus on real-time tracking of Pt click templates in live human cancer cells using Cu-free click reactions that initiate a turn-on fluorescence. Challenges involve the solubility of SPAAC-based fluorophores and the innocence and stability of click handles.

## Pt-azido–based azide–alkyne cycloadditions

The term ‘iClick’ describes cycloaddition reactions between metal azide and metal acetylene groups [34] and has recently been broadened to include the cycloaddition of organic substrates with either a metal azide or metal acetylide [35]. Pt(II) azides undergo cycloadditions with a range of functional groups (e.g. acetylides, isocyanides, isonitriles, nitriles, carbon disulphides and isothiocyanates) [36]. Here, we focus on cycloadditions between azides and acetylene compounds where at least one group is directly Pt bound, reflecting on the potential biological applications of this reactivity.

These reactions enable synthesis of novel complexes and the assembly of multimetallic architectures and polymers. They also provide potential routes for late-stage introduction of sensitive functional groups to Pt complexes and the opportunity for monitoring uptake and subcellular distribution of Pt-azido drugs in cellulo.

Successful Pt-azido–alkyne cycloadditions with organic alkynes typically involve internal alkynes (RC≡CR) because terminal alkynes (HC≡CR) can undergo azide–acetylene ligand exchange at the metal [37]. For Pt-azido–alkyne cycloadditions to occur, activation of the alkyne with a catalyst or with electron-withdrawing groups or strain promotion (SPAAC) is necessary.

### Cu- and Au-mediated azide–acetylene cycloaddition reactions

**Figure 4:**
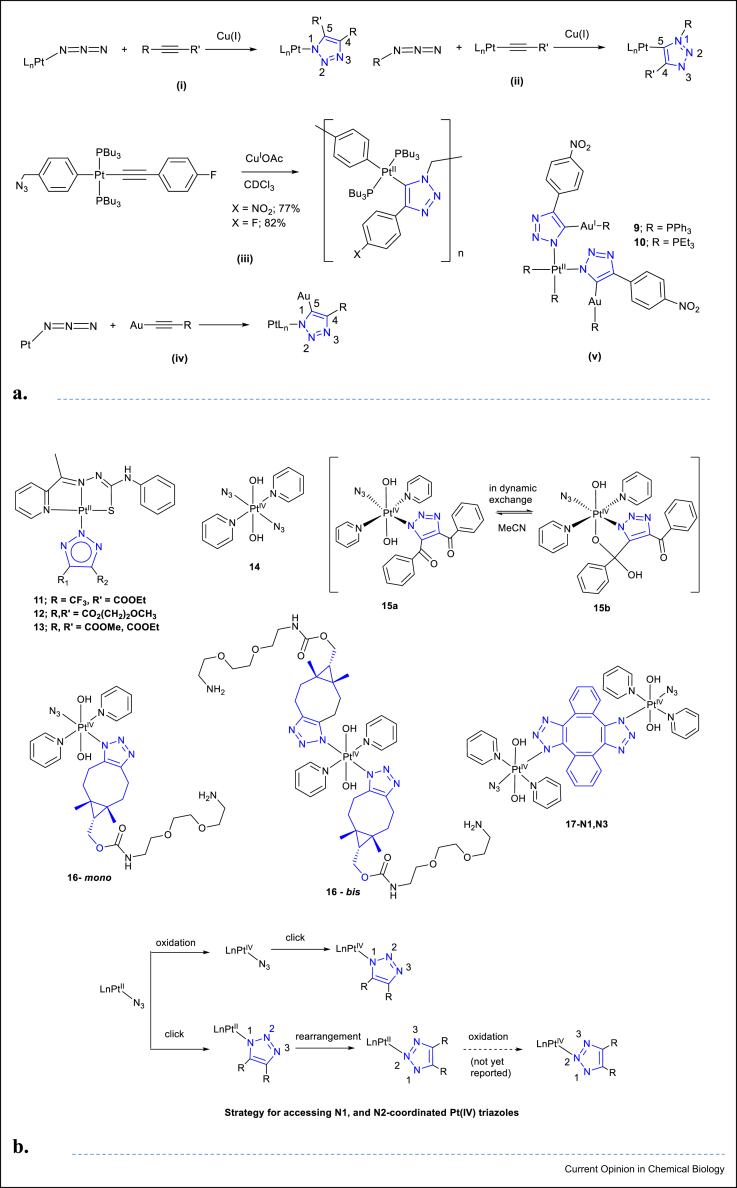
**Pt-azido-based and Pt-alkyne based azide-alkyne cycloadditions. (a)** Cu-catalysed or Au-promoted reactions. General Cu(I)-catalysed synthesis involving (i) Pt-azide and acetylene and (ii) Pt-acetylene and azide; (iii) example synthesis of metallopolytriazolates; (iv) general Au(I)-promoted synthesis of 4,5 trisubstituted 1,2,3-triazolate bridged dimetallic complexes and (v) Pt–Au cycloaddition products, which show some similarity to auranofin [44]. (**b)** Use of electron-deficient acetylenes and strain (SPAAC) to produce Pt(II) and Pt(IV) inorganic click products. Possible different synthetic strategies to access Pt(IV)–N1-coordinated and Pt(IV)–N2-coordinated triazoles. SPAAC, strain-promoted [3 + 2] azide–alkyne cycloaddition.

Typical advantages of CuAAC reactions are the rapid rate of reaction, high yields and the regiospecificity of the resulting product (Figure 4a i,ii). Examples include an in-chain polymerisation of an N_3_-organic spacer-Pt-CC≡R type monomer, giving a novel class of metallopolytriazolates (Figure 4a **iii**) [38]. Au(I) is believed to play a similar role to Cu(I) in traditional CuAAC [38]; if a Au–acetylide is used, cycloaddition with a Pt-azide can proceed in good yield in the absence of a Cu catalyst (Figure 4a **iv**). For example, reaction of *cis*-[Pt^II^(PPh_3_)_2_(N_3_)_2_] with Au^I^PPh_3_(C≡C–C_6_H_4_NO_2_) affords the corresponding Pt(II)/Au(I) heterotrimetallic complex **9**, with two Pt(II) triazole ligands each coordinating a Au(I) phosphine in 92% isolated yield (Figure 4a **v**) [34]; the PEt_3_ derivative **10** which bears some similarity to auranofin — was also synthesised (obtained in 60% yield) [39].

### Electronic- and strain-promoted azide–acetylene click reactions

An advantage of electronic- and strain-promoted methods is biocompatibility; disadvantages include extended reaction times, a restriction on the acetylenes which can be used and if the alkyne is asymmetrical — formation of a mixture of regioisomers.

#### Pt(II) complexes

Although a considerable number of Pt(II) azido cycloadditions have been reported [36], biological investigations of the resultant Pt-triazoles are scarce. A recent example is complex **11** (Figure 4b), which demonstrated an IC_50_ value of 2.4 ± 0.1 μM as compared with 0.9 ± 0.1 μM for cisplatin (GaMG cell line, 72 h contact) [35].

#### Pt(IV) complexes

Judicious ligand choice enables Pt(IV) azides to be nontoxic in the dark; irradiation with visible light induces reduction to Pt(II) species and release of N_3_^•^ and OH^•^ radicals and so on causing potent cytotoxicity in cancer cells. This concept forms the basis for their ongoing development as photochemotherapeutics.[40∗] The first reported cycloadditions of a Pt(IV) azido complex explored the reactivity of complex **14**, Figure 4b, with a range of electron-deficient or strain-promoted acetylenes. Pt(IV) azides are less reactive to azide–acetylene cycloaddition with electron-deficient alkynes than their Pt(II) counterparts, attributed to the higher oxidation state of Pt(IV) which reduces the electron density in the azide ligand. Both Pt(IV) triazolato monoazido and Pt(IV) *bis* triazolato complexes were synthesised through reaction with electron-deficient (1,4-diphenyl-2-butyne-1,4-dione, dimethyl acetylenedicarboxylate) and strained acetylenes (bicyclo[6.1.0]non-4-yn-9-ylmethyloxycarbonyl]-1,8-diamino-3,6-dioxaoctane and dibenzocyclooctyne-amine (**16**-*mono* and **16**-*bis*) [41].

For Pt(IV) triazoles synthesised in this way, there is the potential for additional reactivity with the triazole ligand through the axial (OH) ligand. For complex **15** a reversible interaction with the CO group of the triazole was observed in MeCN (complex **15a**/**15b**) [42]. More recently, studies of the di-Pt(IV) triazole complex **17**-[N1,N3] (Figure 4b) demonstrated that irradiation with visible light (*λ*_irr_ 452 nm) in the presence of 5′-GMP results in the formation of new Pt(IV) and Pt(II) species as well as radical species (N_3_^•^, OH^•^), in H_2_O and cell-free lysate, confirming that monoazido Pt(IV) complexes retain their potential for photocytotoxicity, and click chemistry therefore opens a wide range of late-stage derivation possibilities [43∗∗].

### Post-click reactivity of metal-triazole complexes

Whilst Pt(II) triazoles undergo solvent-dependent N1–N2 isomerisation [35], this has not been observed for Pt(IV)–N1-coordinated triazoles, presumably because of increased kinetic inertness. Different synthetic routes can therefore be envisaged (Figure 4b).

## Conclusions

Azide–alkyne organic click reactions have been used to prederivatise the ligands of Pt complexes, to include targeting agents and fluorophores. A series of Pt complexes containing ligand-based azide or alkyne click handles have also been validated as important templates for functionalisation with complementary partners. Click chemistry–enabled post-treatment labelling of Pt-bound biomolecules (mtDNA, rRNA, tRNA and proteins) and in cellulo tracking of Pt drug surrogates has helped highlight the importance of such approaches in establishing non-nuclear and cytoplasmic effects of Pt-based drugs. The speed of innovations in bio-orthogonal chemistry is anticipated to improve sensitivity and facilitate the real-time tracking of Pt drug surrogates in cellulo. The biological application of cycloadditions involving azides or acetylenes coordinated directly to Pt is in its infancy and ripe for development.

## Conflict of interest statement

Nothing declared.
